# Patient-reported health state utilities in metastatic gastroenteropancreatic neuroendocrine tumours – an analysis based on the CLARINET study

**DOI:** 10.1186/s12955-017-0711-z

**Published:** 2017-06-29

**Authors:** Yang Meng, Grant McCarthy, Anthony Berthon, Jerome Dinet

**Affiliations:** 1 BresMed Health Solutions, North Church House, 84 Queen Street, Sheffield, S1 2DW UK; 2 Ipsen Pharma SAS, Boulogne-Billancourt, France

**Keywords:** Utility, Neuroendocrine tumours, EORTC QLQ-C30, EQ-5D, Mapping, Random-effects model

## Abstract

**Background:**

Gastroenteropancreatic neuroendocrine tumours (GEP-NETs) are rare cancers most often found in the gastrointestinal system or the pancreas. However, patient-reported health state utilities based on clinical trials have not been previously reported in this disease area.

**Methods:**

The CLARINET study collected EORTC QLQ-C30 data from patients in both stable and progressive disease states, although data for the latter were only available during the early stage of progression due to trial design. Using published algorithms, data were mapped to EQ-5D utility values. Random-effects generalised least squares models were used to investigate the impacts of progression status, tumour site and other patient characteristics on mapped utility values.

**Results:**

In total, 1053 observations from 204 patients were mapped to EQ-5D utilities using the McKenzie mapping algorithm. The final random-effects model included age, gender, baseline utility and progression status as covariates; it was not feasible to investigate time-to-death utility due to a limit number of deaths in the CLARINET study. Tumour location (midgut vs pancreas) does not seem to affect utility. However, the difference in utilities based on progression status is statistically significant (*p* < 0.05) in the base case analysis, and the estimated utilities for stable and progressive disease are 0.776 and 0.726, respectively. Furthermore, scenario analyses showed that utility for progressive disease is numerically lower than for stable disease, but this may not be statistically significant in scenarios where alternative Longworth mapping algorithm was used.

**Conclusions:**

Patients with GEP-NETs experience worse utility values in the progressive disease state compared to the stable disease state, based on patient-reported health-related quality of life (HRQL) data from the CLARINET study. The decline of utility in the progressive disease state may be underestimated because progressive HRQL data were only collected shortly after the progression event in the trial. The estimated trial-based utilities can be used in future economic evaluations for GEP-NET treatments and to provide more insights to physicians on patient-reported quality of life outcomes in GEP-NETs.

**Trial registration:**

CLARINET EU Clinical Trials Register Number, 2005–004904-35.

## Background

Gastroenteropancreatic neuroendocrine tumours (GEP-NETs) are rare tumours derived from neuroendocrine cells that can occur anywhere along the gastrointestinal tract and are comprised of a heterogeneous family of neoplasms with a wide and complex spectrum of clinical behaviour [1]. Recent data show an increase of more than 400% in the incidence of this disease, rising from 1.09 per 100,000 population in 1973 to 5.25 per 100,000 population in 2004 [2]. Survival of patients with GEP-NETs depends on stage and histology. Patients with well- and moderately-differentiated distant metastases have a 5-year survival probability of 35%; however, in patients with poorly-differentiated distant metastases, the 5-year survival probability drops to only 4% [2].

Depending on the tumour site, GEP-NETs have traditionally been divided into foregut (oesophagus, stomach, proximal duodenum, liver and pancreas), midgut (distal duodenum ileum, jejunum, ascending colon and proximal two thirds of transverse colon) and hindgut tumours (distal third of transverse colon, descending colon, sigmoid colon and rectum) [1]. Some GEP-NETs produce extra hormones and cause symptoms, and these are called functioning tumours; those that do not produce extra hormones are called non-functioning tumours. GEP-NET treatments can also be broadly divided into treatments to relieve symptoms of excessive hormone production (for functioning tumours) or treatments to control tumour growth and disease progression. Finally, depending on tumour growth and progression, GEP-NET patients can be categorised into those with stable disease and those with progressive disease.

The CLARINET study is a Phase III randomised, double-blind, placebo-controlled, multinational study of the somatostatin analogue (SSA) lanreotide in patients with advanced, well-differentiated or moderately differentiated, non-functioning, somatostatin receptor-positive NETs of Grade 1 or 2 (a tumour proliferation index [on staining for the Ki-67 antigen] of <10%), with tumours originating in the pancreas, midgut or hindgut, or an unknown origin [3]. Altogether, 204 patients were randomised to the lanreotide arm (*n* = 101), and the placebo arm (*n* = 103), and the majority of patients did not have progressive disease at randomisation (195 out of 204). The follow-up period of the trial was 96 weeks. The primary endpoint was progression-free survival, and secondary endpoints included overall survival, safety and health-related quality of life (HRQL) as assessed with the European Organization for Research and Treatment of Cancer QLQ-C30 (EORTC QLQ-C30) and QLQ-GI.NET21. HRQL data were collected at randomisation and at each visit scheduled in the trial. Patients whose disease progressed were withdrawn from the trial, and one additional visit was scheduled 4 weeks after the withdrawal at which HRQL data were also collected. Therefore, HRQL data were collected for both stable disease and progressive disease in the CLARINET study, although the majority of the data were collected while patients’ diseases had not progressed.

EORTC QLQ-C30 cannot be used directly to estimate utility values. However, a number of mapping algorithms have been developed to map from EORTC QLQ-C30 data to EQ-5D-based utilities [4], which can then be used to estimate utility values for different health states. Health technology assessment (HTA) bodies such as the National Institute for Health and Care Excellence (NICE) in the UK also recommended that, in the absence of patient-reported EQ-5D utilities, mapping should be used to convert the available HRQL data to EQ-5D utilities where possible [5].

Utility values, especially health state utility values, are important inputs for assessing the cost effectiveness (cost utility) of new health technologies. In a cost-effectiveness model, the time patients spent in each defined health state (i.e. life years) needs to be weighted by the utility values for that health state to estimate the quality-adjusted life years (QALYs); these are used to calculate incremental cost-effectiveness ratios (i.e. incremental costs versus incremental QALYs) in order to determine the cost effectiveness of the new health technology. For GEP-NETs, the most common health states defined in cost-effectiveness models are stable disease (or progression free) and progressive disease; therefore, it is important that the cost-effectiveness model applies the most relevant utility values to these health states. It will also be helpful to understand how tumours of different sites (e.g. midgut versus pancreas) and time to death (e.g. the last month before death versus more than a month before death) would impact on utilities for GEP-NET patients.

As far as we are aware, there is only one published study performed by Swinburn et al. that reports utility values for GEP-NET patients [6]. In this study, utilities were elicited for health state vignettes describing the HRQL burden associated with receiving therapy for advanced NETs; the vignettes were developed by reviewing literature and conducting in-depth interviews with clinical experts and patients with advanced NETs. A survey of 100 members of the UK general public was performed to value health states using the time trade-off methodology to determine utility values; this showed that stable disease and progressive disease had a utility value of 0.77 and 0.61, respectively, and progressive disease was associated with a significant decline in HRQL. The key limitation of the study is the use of vignettes, which have been criticised for being difficult to establish validity with due to limited comparability to other patient-reported HRQL measures (such as EORTC QLQ-C30 or EQ-5D), which has been criticised for being difficult to establish validity with [7]. In a recent guidance published by the Scottish Medicines Consortium (SMC) for assessing sunitinib for advanced pancreatic NETs with disease progression (SMC 698/11 [8]), utility values of 0.73 and 0.596 were reported for pre-progression and post-progression, respectively, based on converting EORTC QLQ-C30 data to utilities. However, no further details were available on the method used in the SMC guidance. Therefore, patient-reported HRQL data collected in the CLARINET study provide a unique opportunity to investigate health state utility values for GEP-NET patients, something that has not yet been published.

The objectives of this study are to map EORTC QLQ-C30 data collected in the CLARINET study to EQ-5D-based utility values and to apply statistical models on the mapped values to estimate utility values for GEP-NET patients based on progression status (stable disease versus progressive disease), tumour site (midgut versus pancreas), time to death and other factors. The results can provide more insights on patient-reported quality of life outcomes in GEP-NETs, and the estimated utility values can be used in future economic evaluations for HTAs of GEP-NET treatments.

## Methods

### EORTC QLQ-C30 data collected in CLARINET

In the CLARINET study, the EORTC QLQ-C30 questionnaire was completed by patients at randomisation [first visit] and at each subsequent scheduled visit (every 12 weeks in Year 1 [four visits] and every 24 weeks in Year 2 [two visits]) in the trial. For patients who withdrew from the study (including those who withdrew due to disease progression), additional EORTC QLQ-C30 data were also collected 4 weeks after the withdrawal in an early withdrawal visit. No further visits (and consequently HRQL data) were scheduled after this early withdrawal visit.

The follow-up period of the CLARINET study was 96 weeks, and patients whose disease progressed were withdrawn from the trial. Therefore, patients who stayed in the trial for the entire trial period would have seven scheduled visits. These patients were also in the stable disease state during the trial period (otherwise they would have been withdrawn from the trial). For patients who withdrew from the trial, the total number of visits equals the number of scheduled visits before withdrawal plus one early withdrawal visit.

If the reason for withdrawal was disease progression, then EORTC QLQ-C30 data collected at the early withdrawal visit (4 weeks after the last scheduled visit when progression was confirmed) reflects the HRQL for patients with progressive disease. It can also be argued that EORTC QLQ-C30 data collected at the last scheduled visit when progression was confirmed also reflects the HRQL for patients with progressive disease, because the actual disease progression event could occur before the visit. However, it is possible that EORTC QLQ-C30 data collected for patients who withdrew from the trial at the last scheduled visits may overestimate the true HRQL for progressive disease. This is because patients were not aware of the disease progression at these visits (disease progression was confirmed through a central review of an imaging scan from a study visit, and the results were only available after the visits) and patient knowledge of disease progression could have impacted on how the EORTC QLQ-C30 questionnaire was answered. Another consideration is that the utility value for progressive disease should ideally reflect the average utility of the progressive disease health state, which can be very long for GEP-NET patients, whereas the HRQL data collected at the last scheduled visit are likely to be very close to the start of disease progression and hence not reflect the average utility for the progressive disease state. Therefore, in this analysis, the base case assumption is that only HRQL data collected at early withdrawal visits (for patients who progressed) were used for progressive disease. However, in a scenario analysis, both the early withdrawal visits and the last scheduled visits where progression was confirmed were used for progressive disease.

### Mapping EORTC QLQ-C30 to EQ-5D

Eight mapping algorithms linking EORTC QLQ-C30 to EQ-5D were identified from the Health Economics Research Centre mapping database [9–16], but none were specifically designed for GEP-NET patients. Of these eight algorithms, two, one developed by McKenzie et al. [16] and one by Longworth et al. [15], were chosen as appropriate for the mapping in this analysis based on the generalisability to the GEP-NET disease area, external validation, sample size, statistical fit and precedence for use in HTA submissions and for GEP-NETs.

The McKenzie algorithm was developed using data collected from 199 patients with inoperable oesophageal cancer treated with palliative therapies. Data were collected for both the EORTC QLQ-C30 and the EQ-5D, and the algorithm modelled EQ-5D values as a function of the EORTC QLQ-C30 global health status scale, the five function scales and the nine symptom scales [16]. The McKenzie algorithm has been externally validated [9, 17] and widely used in the literature and HTA submissions, including an application for GEP-NET patients in a submission to the pan-Canadian Oncology Drug Review (pCODR) [18].

The Longworth algorithm was developed using pooled data from multiple sources across disease areas including multiple myeloma, breast cancer and lung cancer. Several approaches were attempted to develop the mapping algorithm, with the authors preferring an ordinary least squares model using nine statistically significant single-item scores as well as the age of the patient to map from EORTC QLQ C30 responses to EQ-5D values [15]. The Longworth algorithm was developed more recently and therefore has been less frequently used. However, the algorithm has been externally validated, which suggests that the model preferred by the authors (ordinary least squares model 8) was appropriate [10]. The McKenzie algorithm was chosen as the base case due to its previous use in GEP-NETs; and the Longworth algorithm was used in a scenario analysis.

### Statistical model

The random-effects statistical models were fitted to the mapped EQ-5D utility values and patient characteristics collected in the CLARINET study to investigate utility values based on progression status (stable disease versus progression disease), tumour site (midgut versus pancreas) and treatment group (lanreotide versus placebo), while controlling for relevant patient characteristics such as gender, age, baseline utility (i.e. utility value at initial visit) and tumour progression at baseline (progressed versus not progressed before randomisation). Time to death was not included in the statistical analysis because very few HRQL data were collected close to death (see the results section for more details).

The random-effects model was deemed most appropriate in this analysis because it accounts for the nature of the HRQL data collected in the CLARINET study, specifically repeated utility observations for the same patient over time. The random-effects model accounts for this by clustering and correlation of multiple observations by patient. The random-effects model has been widely used in utility analysis based on patient-level HRQL data collected within clinical trials [19, 20]. All models and analysis were performed using Stata® 12.0 (StataCorp, College Station, Texas, USA).

The following model specifications were tested to inform decisions regarding the inclusion/exclusion of covariates:

• Model 1: age, gender, baseline utility and progression status.

• Model 2: age, gender, baseline utility, progression status and treatment group.

• Model 3: age, gender, baseline utility, progression status, treatment group and primary tumour location.

• Model 4: age, gender, baseline utility, progression status, treatment group, primary tumour location and tumour progression at baseline.

## Results

Patients in the CLARINET study who completed at least one EORTC QLQ-C30 questionnaire were included in the analysis and their EORTC QLQ-C30 data were mapped to EQ-5D-based utilities using the McKenzie and Longworth algorithms. Table 1 shows a summary of the EORTC QLQ-C30 data collected in the CLARINET study and the number of successful conversions for each algorithm. All patients in the CLARINET study had at least one EORTC QLQ-C30 assessment, and a total of 1088 EORTC QLQ-C30 assessments were performed for the 204 patients (5.3 assessments per patient). There were more EORTC QLQ-C30 assessments per patient in the lanreotide arm compared to the placebo arm (5.5 versus 5.1 per patient) because of the longer progression-free survival of patients in the lanreotide arm.

**Table 1:** Summary of EORTC QLQ-C30 data collected in the CLARINET study and the number of successful mappings to EQ-5D-based utility values

		All patients	Lanreotide	Placebo
ITT population		204	101	103
Total assessments		1088	559	529
Assessments per patient	Mean	5.5	5.1	5.5
	Median (Min–Max)	7 (1–8)	5 (1–7)	7 (1–8)
Mapped to EQ-5D using McKenzie algorithm [16] (N, %)		1053 (96.8%)	540 (96.6%)	513 (97.0%)
Mapped to EQ-5D using Longworth algorithm [15] (N, %)		1041 (95.7%)	530 (94.8%)	511 (96.6%)

Key**:** *ITT* intention to treat

Of the 1088 EORTC QLQ-C30 assessments, 1053 (96.8%) and 1041 (95.7%) assessments were mapped to EQ-5D utility values using the McKenzie and Longworth algorithms, respectively. The unsuccessful mappings were due to the EORTC QLQ-C30 questionnaire being only partially completed and/or some patient characteristics required by the algorithms being missing. The numbers of unsuccessful mapping differ between the two algorithms because the McKenzie algorithm uses 15 domain scores of the EORTC QLQ-C30, whereas the Longworth algorithm uses nine statistically significant single-item scores from the EORTC QLQ-C30 questionnaire as well as the age of the patient.

**Fig. 1:**
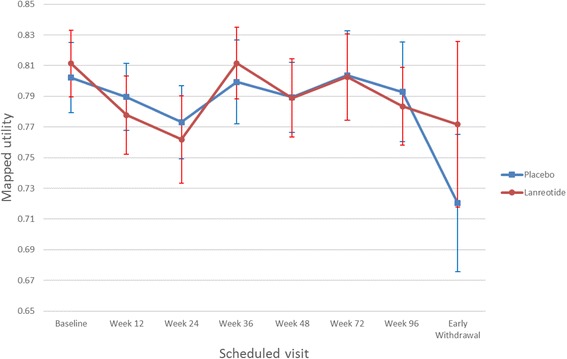
EQ-5D-based utility values (McKenzie algorithm) by visit in CLARINET

Table 2 presents the descriptive statistics of mapped EQ-5D-based utility values by mapping algorithm, treatment arm, tumour site and progression status (including the numerical difference between stable and progressive diseases). The descriptive statistics showed that the two mapping algorithms provided broadly similar EQ-5D-based utility values (e.g. overall mean EQ-5D-based utility values are 0.79 and 0.80 using the McKenzie and Longworth algorithms, respectively). The descriptive statistics also suggested similar utility values for tumours located in the midgut and pancreas and that utility values for progressive disease are numerically lower than for stable disease. The descriptive utility values between lanreotide and placebo were also similar for the midgut and pancreas and for stable disease; however, the lanreotide arm had numerically higher utility values for progressive disease (see Table 2). Fig. 1 shows the mapped EQ-5D-based utilities (mean and confidence interval) by treatment arm and by each visit in the CLARINET study (seven scheduled visits plus the early withdrawal visit). The figure shows that the utility values remain broadly similar during the trial period for both treatment arms, and that utility values at the early withdrawal visit appear lower than utility values at scheduled visits for the placebo arm (not so much for the lanreotide arm). It also shows there were no significant differences in utility values between the lanreotide and placebo arms across all visits. Please note, these observations were based on descriptive statistics and did not account for repeated HRQL assessments for patients or for patient characteristics that could impact on utilities; therefore, they are preliminary findings that need to be tested and confirmed by formal statistical models (i.e. the random-effects model in this analysis).

**Table 2:** Mapped EQ-5D utilities by mapping algorithm, treatment arm, tumour site and progression status

	McKenzie algorithm [16]						Longworth algorithm [15]					
	Overall		Lanreotide		Placebo		Overall		Lanreotide		Placebo	
	Mean (SE)	Sample size	Mean (SE)	Sample size	Mean (SE)	Sample size	Mean (SE)	Sample size	Mean (SE)	Sample size	Mean (SE)	Sample size
All patients	0.79 (0.01)	1053	0.79 (0.01)	540	0.79 (0.01)	513	0.80 (0.01)	1041	0.80 (0.01)	530	0.80 (0.01)	511
Midgut	0.77 (0.01)	412	0.79 (0.01)	193	0.76 (0.02)	219	0.79 (0.01)	407	0.80 (0.01)	192	0.78 (0.01)	215
Pancreas	0.80 (0.01)	430	0.79 (0.02)	210	0.81 (0.01)	220	0.80 (0.01)	426	0.80 (0.01)	204	0.80 (0.01)	222
Stable disease	0.79 (0.01)	997	0.79 (0.01)	522	0.79 (0.01)	475	0.80 (0.01)	986	0.80 (0.01)	513	0.80 (0.01)	473
Progressive disease	0.72 (0.04)	56	0.77 (0.05)	18	0.70 (0.04)	38	0.75 (0.02)	55	0.78 (0.04)	17	0.74 (0.03)	38
Utility decrement	−0.07		−0.02		−0.10		−0.05		−0.02		−0.06	

Key: *SE* standard error

One aim of the analysis was to explore the impact of time to death on the utilities of patients with GEP-NETs. The estimation of time to death requires death events and the knowledge of the timing of death. Only four deaths occurred in the CLARINET study, with a further 32 deaths occurred in the CLARINET extension study (which followed a subset of patients in the original CLARNET trial) with majority of deaths occurred long after the end of the original CLARINET study. Please note that no HRQL data were collected in the CLARINET extension study and only the timing of deaths occurred in the CLARINET extension study was used in this analysis. Among the EQ-5D-based utilities mapped from EORTC QLQ-C30 in the CLARINET study, a total of 156 mapped EQ-5D-based utilities were identified that related to these 36 patients who experienced death events in either the original CLARINET study or the CLARINET extension study. Time to death for each of these EQ-5D-based utilities was calculated as the difference between the timing of death and timing the HRQL data was collected in the CLARINET study. As majority of deaths occurred in the CLARINET extension study and long after the end of the CLARINET study, majority of time to death statistics calculated were long. Specifically, there were only three EQ-5D-based utility values that were observed within 60 days prior to death (one within 30 days and two between 30 and 60 days), a further 20 EQ-5D-based utility values between 60 and 360 days prior to death, and with the rest of EQ-5D-based utility values more than 360 days prior to death. Literature suggests the impact of time to death on HRQL is important to consider, especially in the weeks preceding death [19], and clinical experts suggest that utility for GEP-NET patients may decrease significantly in the 30–60 days prior to death. Unfortunately, the limited time to death HRQL data available from the CLARINET study (even with the additional death events identified from the CLARINET extension study) means this analysis is not feasible; hence, time to death was not further explored in the statistical models.

Four model specifications with different lists of covariates were tested by the random-effects models. Based on the Akaike information criterion (AIC) and the Bayesian information criterion (BIC), the simpler Model 1 (which includes covariates of age, gender, baseline utility and progression status) had the best AIC and BIC and was therefore chosen as the base case. Table 3 presents the detailed model results for the base case Model 1 when EQ-5D-based utility values mapped from the McKenzie algorithm were applied. It shows that progressive disease has significantly lower utility values compared to stable disease, with a mean estimated utility decrement of 0.0495 (standard error 0.018; *p*-value 0.005). Age was also found to be negatively linked to utilities (*p*-value 0.03), whereas gender was not shown to have a significant impact on utilities (*p*-value 0.77).

**Table 3:** Base case random-effects model results (Model 1)

	Coefficient	Standard error	*p*-value	95% CI	
Age	−0.0014867	0.0006949	0.032	−0.0028487	−0.0001247
Gender	−0.0043254	0.0148942	0.772	−0.0335175	0.0248667
Baseline utility	0.8060277	0.0346037	0.000	0.7382056	0.8738497
Progression status	−0.0495048	0.0176029	0.005	−0.0840059	−0.0150038
Constant	0.2279264	0.052085	0.000	0.1258417	0.3300111

Key: *CI* confidence interval

If we focus on the impact of progression status on utility value, the estimated utility decrements across all tested model specifications were very similar when the McKenzie algorithm was applied (ranges from 0.0495 to 0.050) and when the Longworth algorithm was applied (ranges from 0.0284 to 0.0292) (see Table 4 for details). The utility decrement for progressive disease estimated in models based on the McKenzie algorithm is bigger (also statistically significant when *p* < 0.05) than models based on the Longworth algorithm (also not statistically significant when *p* > 0.05), which is consistent with the findings based on the descriptive statistics.

**Table 4:** Summary of estimated utility decrements for progressive disease versus stable disease

Model	McKenzie algorithm [16] (base case)				Longworth algorithm [15]			
	Coefficient	95% CI		*p*-value	Coefficient	95% CI		*p-value*
1 (base case)	−0.0495	−0.0840	−0.0150	0.005	−0.0284	−0.0586	0.0019	0.066
2	−0.0499	−0.0845	−0.0154	0.005	−0.0292	−0.0595	0.0011	0.059
3	−0.0496	−0.0842	−0.0150	0.005	−0.0288	−0.0591	0.0015	0.062
4	−0.0500	−0.0846	−0.0153	0.005	−0.0291	−0.0594	0.0013	0.061

Key: *CI* confidence interval

Finally, the estimated random-effects models (base case model and models for scenario analyses) were used to estimate the absolute utility values for stable disease and progressive disease based on the mean patient characteristics observed in the CLARINET study, including the mean age (62.1), mean proportion of males (52%) and mean baseline utility (0.80). The estimated base case utility values for stable disease and progressive disease were 0.776 and 0.726, respectively. Table 5 compares the utility values for stable disease and progressive disease in three scenario analyses:

- McKenzie algorithm and alternative assumption for categorising progressive disease HRQL data;
- Longworth algorithm and base case assumption for categorising progressive disease HRQL data; and
- Longworth algorithm and alternative assumption for categorising progressive disease HRQL data.

**Table 5:** Comparison of utility values for stable and progressive health state

	Base case utilities	Scenario 1	Scenario 2	Scenario 3	Swinburn 2012 [6]	SMC 698/11 [8]
Stable disease	0.776	0.780	0.793	0.796	0.771	n/a
Progressive disease	0.726	0.732	0.764	0.764	0.612	0.73^a^

Note:^a^, 0.73 relates to pre-progression for patients with pancreatic neuroendocrine tumours with disease progression (and therefore corresponds to progressive disease in this analysis) and the utility value for these patients post-progression is 0.596

The comparison shows that utility values estimated based on the Longworth algorithm are higher than values based on the McKenzie algorithm, and the estimated utility decrements are higher using the McKenzie algorithm compared to the Longworth algorithm. The utility values estimated by Swinburn et al. [6] and reported in the SMC guidance 698/11 [8] are also presented in Table 5 for comparison.

## Discussion

No studies are presently available analysing patient-reported health state utilities in metastatic GEP-NETs. Existing literature on GEP-NETs utilities was either based on data collected from the general public [6] or did not report details of how the reported utilities were estimated [8]. The CLARINET study provides a unique opportunity to investigate utility values for GEP-NET patients using patient-level HRQL data collected in a Phase III trial. The patient-level HRQL data included up to seven scheduled HRQL assessments for each patient and an early withdrawal assessment for patients who withdrew from the trial (e.g. due to disease progression), and they provide a range of patient characteristics that can be included in the statistical analysis. The base case random-effects model used 1053 mapped EQ-5D-based utility values, and the best fit model includes age, gender, baseline utility and progression status as covariates.

The base case model shows that progressive disease is associated with significantly worse utility compared to stable disease (*p*-value 0.05), and the estimated utility values for stable and progressive disease using mean patient characteristics from the CLARINET study are 0.776 and 0.726, respectively. The estimated utility value for stable disease from this analysis is close to the reported utility for stable disease by Swinburn et al. (0.771) [6], but the estimated utility value for progressive disease from this analysis is higher than the value reported in the literature (0.612). The method used by Swinburn et al. was based on vignettes and health states valued by a UK general population sample of 100; therefore, the utility values may not be directly comparable due to the different methodologies used. Nevertheless, the comparison with Swinburn et al. provides some assurance regarding the utility value for stable disease. The only other GEP-NET utility values available in the literature are from the guidance published by the SMC for sunitinib [8], which reported utility values of 0.73 and 0.596 for pre-progression and post-progression respectively for advanced pancreatic NET patients with disease progression. The sunitinib trial compared sunitinib plus best supportive care versus best supportive care, where best supportive included the use of SSAs if required for symptomatic control. Given the patients of interest in the sunitinib trial were those whose disease had progressed while the patients of interest in the current analysis are those whose disease had not progressed, the reported pre-progression utility (0.73) should be comparable with the progressive disease utility (0.726) estimated in the current analysis, which are very similar. The only information available regarding the method was that the utility values were converted from EORTC QLQ-C30 data from the sunitinib trial. Although no detailed methods were given in the SMC guidance (e.g. mapping algorithm, how utility values were derived), the general methods used are similar (i.e. convert trial-based EORTC QLC-C30 data to utilities), and the estimated progressive disease utility value in the current analysis appears comparable.

The estimated utility values in this analysis are also broadly in line with utilities reported in the wider population of cancer patients with similar disease progression status (i.e. progression free and post-progression for advanced stage cancers). For example, in recent NICE HTA submissions, utility values of 0.8018 and 0.7277 were reported for progression free and post-progression, respectively, for advanced melanoma [21], and 0.763 and 0.675 for progression free and post-progression, respectively, for advanced non-small cell lung cancer [22]. These reported utilities were all based on patient-level EQ-5D data collected in clinical trials.

The estimated utility decrement for progressive disease compared to stable disease in this analysis appears to be small (0.0495) compared to an absolute decrement of 0.159 based on the study by Swinburn et al. [6]. There are few potential explanations. Firstly, much fewer HRQL data were collected for progressive disease in this analysis (56 out of the 1053 mapped utility values related to progressive disease collected in the early withdrawal visits); therefore, estimates for progressive disease utilities are less robust and certain. Secondly, progressive disease utilities are only available for one visit (i.e. early withdrawal visit) for patients whose disease progressed in the CLARINET study. Therefore, the progressive disease utility values estimated in this analysis may not represent the average utility for GEP-NET patients who have progressive disease, which can last for a long period of time. Clinical experts suggested that utility may decrease over time during the progressive disease state, especially shortly before death. Therefore, it is likely that the progressive disease utility estimated in this analysis would underestimate the true utility for progressive disease. However, a statically significant utility decrement for progressive disease compared to stable disease was found in this analysis (*p*-value 0.05), and this strengthens the common hypothesis that patients with GEP-NETs have lower utilities in progressive disease states compared to stable disease states.

Given the above, this study strengthens the evidence base for HRQL and utilities experienced by GEP-NET patients and provides physicians with insights into patient-reported HRQL outcomes. The significant utility decrement for progressive disease compared to stable disease found in this analysis further supports the benefits of GEP-NET treatments with SSAs such as lanreotide that delay disease progression (even without prolonging survival) due to the better HRQL for patients in stable disease states compared to progressive disease states. The utility values estimated in this analysis can be used in future economic evaluations for GEP-NET treatments, especially for patients with non-functioning midgut or pancreatic NETs of Grade 1 or 2 without disease progression.

There are a number of limitations of the analysis. EQ-5D data were not collected in the CLARINET study; therefore, mapping algorithms were required to map HRQL data collected in the CLARINET study to EQ-5D-based utilities. Although mapping is widely used in economic evaluations, and in particular, many mapping algorithms between the EORTC QLQ-C30 and EQ-5D have been developed and applied in the literature, there is no specific mapping algorithm for GEP-NET patients, and the use of mapped utility values (rather than directly reported utility values) is less than ideal and will introduce a degree of uncertainty regardless of how suitable the mapping algorithm is. In this analysis, the McKenzie algorithm used in the base case analysis tends to overestimate mild health-state utilities and, in extreme cases, to predict utilities >1. In this analysis, 12.8% of all mapped utility values generated using the McKenzie algorithm are >1 with an average utility value of 1.03 (range 1–1.06). The issue of predicting unity values > 1 has been previously reported [17], but there is no consensus of how to deal with this problem, and some studies have used the unadjusted mapped utility data (i.e. not adjusting mapped utilities that are >1 to 1) [17]. This analysis used the unadjusted mapped utility data, and the impact is expected to be small due to the small proportion of problematic mapped utilities and because the problematic mapped utilities are very close to 1. The Longworth algorithm did not result in mapped utilities >1 in this analysis.

The analysis was performed using data from the CLARINET study, which mainly recruited patients with stable disease and with well-differentiated or moderately differentiated, non-functioning GEP-NETs. Therefore, the findings and estimated utilities from this analysis is most relevant to non-functioning GEP-NETs patients whose disease has not progressed and who initially has well-differentiated or moderately differentiated disease. For patients whose disease has already progressed, the progressive disease utility value estimated in this analysis should be relevant, The results of the analysis may not be applicable to functioning patients, patients initially diagnosed with poorly differentiated disease or patients with progressive disease who have further progressed. The CLARINET study has a maximum follow-up period of 2 years for collecting health utility data. Given majority of patients in the CLARINET study were recruited during the stable phase of the disease and the slow progression nature of the disease, the relatively short follow-up period of the CLARINET trial limits the amount of health utility data collected for patients who have progressed. Another limitation is that it was not possible to carry out time to death analysis due to a lack of deaths and a small sample size of HRQL data shortly before death.

Given the limitations of this analysis, it is suggested that generic HRQL instruments such as EQ-5D can be used in future GEP-NET trials to avoid the use of mapping algorithms for utility analysis. The development of mapping algorithm specific to GEP-NETs patients will also be useful to better map HRQL data collected in existing GEP-NETs trials. The collection of more HRQL data post-disease progression in future trials with longer follow-up period would also be very helpful to help estimate robust utility values for the disease progression state and time to death utilities. When such data are available, future research can estimate more robust utility values for GEP-NET patients and the results from this analysis can be compared against and validated. Another area for future research is for estimating patient-reported health utilities for functioning and poorly differentiated GEP-NET patients. Finally, future economic modelling studies can be performed to evaluate the cost-effectiveness of competing treatments for treating stable GEP-NETs patients using the estimated health utilities from this study.

## Conclusions

Patients with GEP-NETs experience worse utility values in the progressive disease state compared to the stable disease state, based on patient-reported HRQL data from the CLARINET study. The decline of utility in progressive disease may be underestimated as progressive HRQL data were only collected shortly after the progression event in the trial. The estimated trial-based utilities can be used in future economic evaluations for GEP-NET treatments and to provide more insights to physicians on patient-reported quality of life outcomes for patients with GEP-NETs.

## Abbreviations

AIC: Akaike information criterion
BIC: Bayesian information criterion
EORTC QLQ-C30: European Organization for Research and Treatment of Cancer Quality of Life Questionnaire-C30
EORTC QLQ-GI.NET21: European Organization for Research and Treatment of Cancer Quality of Life Questionnaire - gastrointestinal neuroendocrine tumour specific module
EQ-5D: EuroQol five-dimensions
GEP-NETs: Gastroenteropancreatic neuroendocrine tumours
HRQL: Health related quality of life
HTA: Health technology assessment
NICE: National Institute for Health and Care Excellence
pCODR: pan-Canadian Oncology Drug Review
QALY: Quality adjusted life-year
SMC: Scottish Medicines Consortium
SSA: Somatostatin analogue
